# How Did Prenatal Education Impact Women’s Perception of Pregnancy and Postnatal Life in a Romanian Population

**DOI:** 10.3390/medicina57060581

**Published:** 2021-06-07

**Authors:** Anca Maria Balasoiu, Octavian Gabriel Olaru, Romina Marina Sima, Liana Ples

**Affiliations:** 1 Department PhD, IOSUD, “Carol Davila” University of Medicine and Pharmacy, 020021 Bucharest, Romania; anca.balasoiu@drd.umfcd.ro; 2 “Bucur” Maternity, “Saint John” Clinic Emergency Hospital, 042122 Bucharest, Romania; romina.sima@yahoo.es (R.M.S.); liana.ples@umfcd.ro (L.P.); 3 Department of Obstetrics and Gynecology, “Carol Davila” University of Medicine and Pharmacy, 020021 Bucharest, Romania

**Keywords:** prenatal education, breastfeeding, newborn care

## Abstract

*Background and Objectives:* Prenatal education represents an important part of maternal prenatal care in Western countries. In Romania, prenatal education is of recent interest but there is no official information about prenatal courses and their impact on prenatal care and patients in Romania. *Material and methods:* A prospective study based on the STROBE statement was designed in order to assess the prenatal education delivered in our unit. The study group included women who gave birth at Bucur Maternity, “Saint. John” Hospital, Bucharest, Romania and attended the prenatal courses, compared with a control group (women who gave birth in our unit but did not attend the prenatal lecture). Patients’ perception about the impact of prenatal education was collected by applying a questionnaire. *Results:* The analysis included 89 women who fulfilled the questionnaire online. In our study, 62 women (69.7%) attended the prenatal education classes and represented the study group while 27 women (30.3%) constituted the control group. Women who attended the prenatal lecture recognized the utility of the topic regarding newborn care (90.3%), while women from the control group did not consider it useful (*n* = 55.6%), χ2 = 18.412, *p* < 0.001. Patients from the study group admitted the importance of the topics (93.5%) from the lectures about breast feeding, while the percentage of these women from the control group is significantly lower (55.6%) χ2 = 27.867, *p* < 0.001. *Conclusions:* The benefits of prenatal education were recognized by women who attended the prenatal lecture, while women who did not participate underestimated the utility of the topics. Further actions are required to inform mothers about the necessity of antenatal education.

## 1. Introduction

Prenatal education represents an important part of maternal prenatal care in Western countries, whereas in Eastern European communities these classes are not common [1]. The main purpose of prenatal education is to prepare the mother and or the couple for new-born care, but also to familiarize the expectant couples with the main events that will occur during pregnancy and the birthing process. There are few data regarding the incidence of prenatal education in different countries, but in Canada the attendance of prenatal education is about 33% [2] and in Australia it is 84% for nulliparous women [3]. There is no official information about the extent of prenatal education classes and their degree of attendance in Romania. Culturally, prenatal education is provided through the shared experiences of families or relatives (mostly in the rural or low-income or poorly educated population) or through the internet or shared media groups (for younger and more media dependent people). The quality of the content, the way of delivering information, and the impact of those prenatal ‘educational’ methods are not always reliable or useful. In our unit, we have 6 years’ experience in providing prenatal classes for expectant couples, and the aim of the study is to assess the quality of those programs, the impact of the classes on improving the postnatal care of the babies, the general and mental health of the mothers, and to provide relevant information for decisional factors in order to extend and standardize prenatal education in Romania based on the specific needs of our population. In our country, there are prenatal lectures but nothing is standardized. In different maternity wards there are midwives or clinicians who are involved in such activities. The topics are chosen at their own will based on their own experience. In the present study, we wanted to evaluate the opinion of women who attended the lectures regarding the topics by comparing them with women who did not participate in prenatal education.

## 2. Materials and Methods

A prospective study based on the STROBE statement was designed. The study group included women who gave birth at Bucur Maternity, Saint. John Hospital, Bucharest, Romania and attended the prenatal classes. The study group was compared with a control group that included patients who gave birth at Bucur Maternity but did not attend the prenatal classes. Patients’ perception about the impact of prenatal education were collected by applying a questionnaire. Prior to research, the study protocol was approved by the Ethics Committee of “St. John” Hospital 27247/16 December 2019.

The participants were recruited directly from the parents attending the prenatal classes. The questionnaire was addressed only to women who gave birth in our unit because they attended the prenatal lectures that had the same lecturer. We wanted to exclude the heterogeneity caused by different lecturers and the subjectivity of teaching.

The main inclusion criteria were represented by pregnant women who gave birth in our department, aged over 18. The questionnaire is not a standardized one, but it was created by our research team. Although the questionnaire was originally intended to be completed 6–8 weeks after birth, it was also completed by women who did not meet this requirement. Also, as there was no standard for completing the child’s age, the month was used as a unit of measurement in the calculations performed. The parameters included were: patient age, residence in urban/rural areas, personal studies, and questions about the role of prenatal care. The exclusion criteria from the study were represented by patients who did not consent to participation, who were under the age of 18, or who did not fulfill all prenatal classes. Considering the context of the COVID-19 pandemic in Romania, the questionnaire was carried out online.

The data were analyzed using SPSS version 25.0 (statistical packages for social sciences). Pearson’s correlation and two-sided *p* values of <0.05 were considered statistically significant. The risk of bias is represented by the reduced number of patients and the subjectivity of the persons that completed the questionnaire.

## 3. Results

The analysis included 89 women who completed the questionnaire online between 25 June 2020–8 August 2020. The information about the total group are as follows: regarding their age, the overall distribution was: 43 women (48.3%) aged 30–35 years, 23 women (25.8%) aged 25–30 years, 14 women (15.7%) aged 35–40 years, 6 women (6.75) aged between 20–25 years, 2 women (2.2%) who were over 40 years old, and1 woman (1.1%) in the age group 18–20 years.

The characteristics of the entire group are: an increased frequency of women with university studies, representing more than three quarters of respondents (76.4%, *n* = 68 women), followed by those with high school education (13.5%, *n* = 12 women), post-secondary education (5.6%, *n* = 5), and by women with postgraduate studies (master) (4.5%, *n* = 4). Thus, 80.9% of the mothers who completed the questionnaire (*n* = 72) had a university education (university + postgraduate) and 19.10% (*n* = 17) did not (high school + post-high school). The results indicated a high participation in classes for women living in urban (88.8%) areas and only 11.2% were rural residents.

The questionnaire was completed by mothers whose children were aged between 3 weeks and 4 years, who had an average age of 8.8 months (approximately 8 months and 3 weeks) (standard deviation: 9.64, CI: (6.79; 10.85)). Half of the women who completed the questionnaire have children under5 months old. The majority of mothers (74) who completed the questionnaire had only one child, representing 83.1% of the women in the whole group, while16.9% (15 women) had more than one child. Within the entire group, 71 women (79.8%) gave birth by cesarean section, while only 18 (20.2%) gave birth naturally.

**Figure 1:**
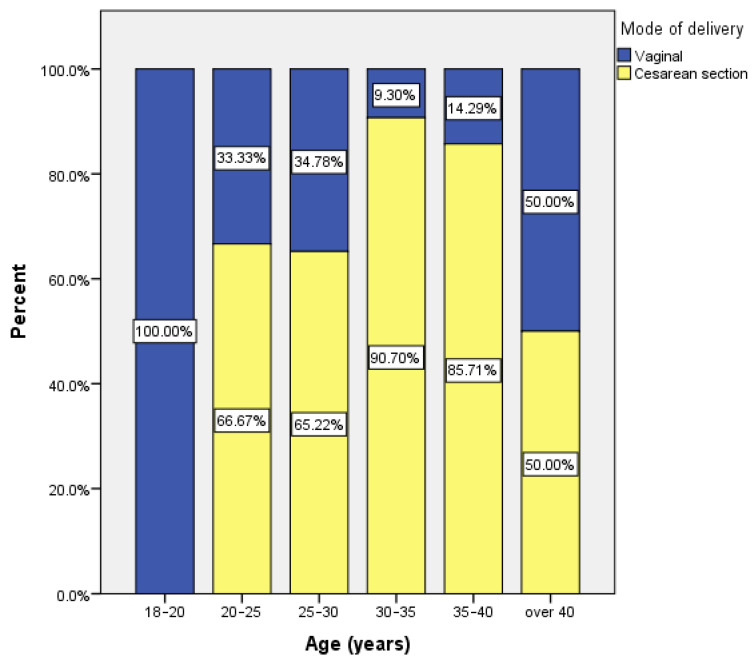
Patient distribution by age and delivery method.

Depending on the age of the women in the whole group, the following aspects are noted. Only a third of women between the ages of 20 and 30 gave birth naturally (Figure 1).

Over 90% of women in the 30–35 age category, and over 85% of those aged 35–40 gave birth by cesarean section. There were no statistically significant associations between the method of birth and the level of education, the place of origin, or the parity.

In our study, 62 women (69.7%) attended the prenatal education classes and represented the study group, while 27 women (30.3%) did not participate and constituted the control group.

**Figure 2:**
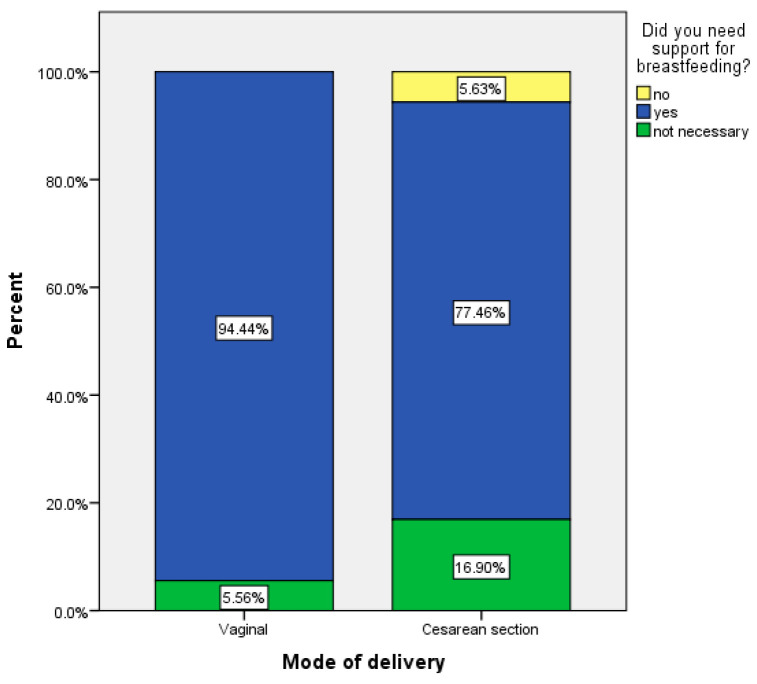
Patients who required support for breast feeding according to delivery mode.

Women who attended the prenatal lecture acknowledged the utility of the topic regarding newborn care (90.3%) while women from the control group did not consider it useful ((*n* = 55.6%), χ2 = 18.412, *p* < 0.001 (Table 1)). Patients in the study group admitted the importance of the topics from the lectures about breast feeding and newborn feeding generally, while the percentage of those women from the control group is significantly lower ((55.6%) χ2 = 27.867, *p* < 0.001). Moreover, patients who delivered by the vaginal method more often required support for breastfeeding (94.44%) compared with those who delivered by caesarean section (77.46%) (Figure 2).

**Figure 3:**
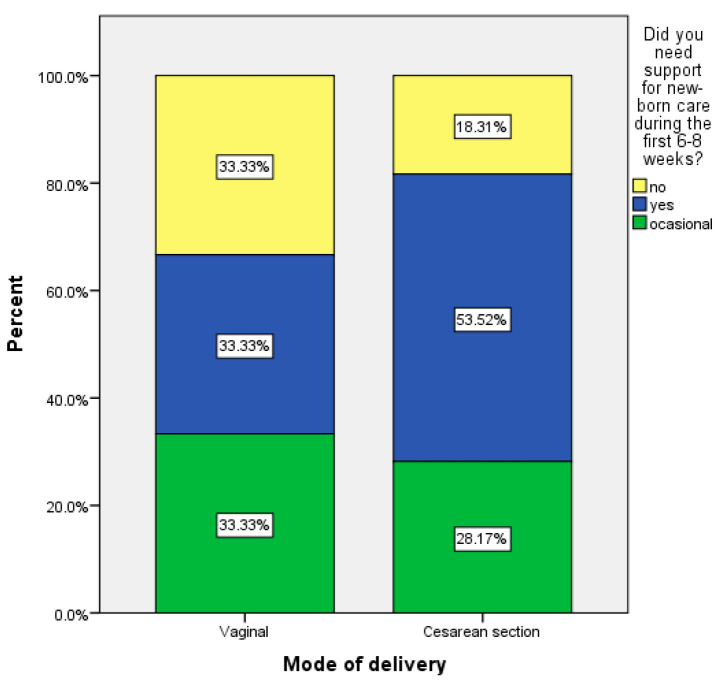
Patients who required support for newborn care according to delivery mode.

Moreover, women from the study group considered the information about birth preparation very important, while women from the control group who found the same topic useful constituted only a small percentage ((14.8%) χ2 = 22.451, *p* < 0.001). The topic about mother alimentation and hygiene is also more interesting from the point of view of women who attended the prenatal lectures compared with the control group (χ2 = 3.673, *p* = 0.045). Also, women who delivered by caesarean section required more frequent support for newborn care (53.2%) compared with women who delivered by the vaginal method (33.3%) (Figure 3).

Women in the study also describe that their partner was interested in the topic from the prenatal lecture, but patients who did not attend the lecture did not consider the course useful for them. The difference is not statistically significant. The same situation is found in the necessity of mother help during the postpartum period, with no significant differences between the two groups.

## 4. Discussion

Several issues can be discussed when we talk about prenatal education in Romania. As we can observe from our study, there is a considerable difference between the analyzed groups. Women who attended the lectures considered that the following lot of topics were important: new-born care, birth preparation, alimentation, hygiene, and partner involvement. The mothers who did not participate in prenatal lectures dismissed the importance of this type of education. We may say that this aspect represents a problem from many points of view. Mothers who did not take the prenatal lecture are not interested in the subject and may miss useful information about newborn care. Another aspect is that they may influence other mothers with their ideas. Our findings from this study are original because we did not identify similar reports in the literature.

The study reveals that not only did the women who attended the course find it useful (93.5%), but more than half (55.6%) of those who did not access prenatal education classes also did so. The latter category found that at least some topics related to newborn care are useful (55.6%). This shift of perception could lead to an increased interest in attending prenatal classes in subsequent pregnancies.

Prenatal education may be a key factor for expecting couples regarding the quality of the prenatal care in pregnancy and the addressability to medical services [4]. Even if there are a variety of reports and a lot of publicity regarding prenatal courses, the majority of the studies observed that prenatal education is a health promotion method and it may be considered the core of prenatal public policies [5]. Studies have proven that prenatal education is an effective tool to reduce labor and delivery related anxiety and increase the partners’ involvement in the neonatal period [6]. In Romania, there are many prenatal education providers, most of them connected to private or public health care facilities (hospitals, maternities), but also patient education association. There is no standard for prenatal education, and different providers found various methods to increase the future parents’ interest in those activities. Lately, they have opted for online prenatal education, alone or combined with group “face to face” prenatal classes. The impact or the effectiveness of each educational mode has not been systematically evaluated, and even if there are internal audit feedback reports they have not been published and therefore cannot be evaluated. Some prenatal educational providers are concerned with improving their efficiency and visibility and are rethinking couching methods based mainly on online education.

There are authors who consider prenatal education to have limited efficiency because of the heterogeneity of delivery modes, limiting its contribution [7], especially online prenatal education [8]. However, online education may be the only solutionfor fulfilling the needs of certain users in specific conditions (such the pandemic period), and it may improve [9] the accessibility of prenatal lectures [10].

A Canadian study was reported that tried to evaluate the impact of online prenatal education on health determinants. It makes an important reference to knowledge acquisition and online prenatal education as a new technological service delivery model. It will compare the online courses with the absence of group prenatal education in certain health services center settings. Parameters based on complementarity of group and online prenatal education and those related to a community setting are expected to offer important information about perinatal service networks. Moreover, this is underscored by the fact that involving future fathers or partners is common, even if their contribution in the perinatal period is strongly recommended. The results of that study will try to show how group and online prenatal education can play a key role in the wellbeing of a family [11].

Another issue is related to the topics that are of interest to the expecting couples, which can be different from the ones that the providers think proper to offer. Labor and delivery, baby care and safety, breastfeeding, and maternal mental health are topics with different weight when it comes to the public interest, and therefore prenatal education should identify and individualize those points of interest for each couple and group. Delivering the same information in large groups with different interests can negatively impact the general benefits of the prenatal education.

Prenatal education represents an essential component of maternity care because it facilitates support and makes it easier to get information from health care providers. It adds partner involvement, decreases anxiety about labor and birth, and creates a friendly environment where questions can be addressed without hesitation [12]. This type of education is crucial in the third trimester of pregnancy, when major physiological, but also psychological, changes develop and women face many decisions. A fear of childbirth brings risks to a healthy adjustment from pregnancy through birth and into the postpartum period. It is caused by low childbirth self-efficacy, the use of pain medication, obstetric interventions in labor [13], and the risk of postpartum depression. Prenatal education courses represent a tool by which pregnant women are informed about coping strategies with labor pain, even if childbirth education has reduced the efficacy of childbirth fear removal and in isolated cases it may augment child related fear. It is recognized that with more than 3.9 million births in the United States per year, accessible methods for addressing childbirth fear and birth related pain are critically required. Mindfulness training provides a recent and promising strategy for educating women for childbirth [14].

Prenatal education is destined to teach women and their relatives about the psychological changes of pregnancy, what to expect during the prenatal period, and how to manage labor, birth, and newborn care [15]. A Cochrane systematic review conducted by Gagnon and Sandall in 2007 [16], and recently updated by Brixval et al. [17], evaluated the impact of individual or group antenatal education and concluded that the effect of antenatal education on health and psychosocial outcomes is unclear. A more recent systematic review evaluating the effect of antenatal education on labor and birth highlighted positive effects that might include less anxiety, fewer false labor admissions, and more partner involvement. In Canada, the first national study of women’s childbirth experiences proved that 65.6% of Canadian women were the beneficiaries of prenatal classes in their first pregnancies [2]. In the same study, it was observed that women living in a household at or below the low-income cutoff were less likely to attend classes (24.1%, 95% CI 21.6–26.7) than women living in a household above the low-income cutoff (34.7%, 95% CI 33.5–35.9) [2]. Significant differences appeared also between urban versus rural maternity care) [18].

Other studies concerned with women’s views and pregnancy interventions reported in Argentina and Australia found that prenatal education would enhance women’s access to high-quality, pregnancy-related information [19]. Recent studies included hyperlinks to pregnancy and parenting groups for women to have access to health care professionals. Future iterations of the program may bring advantages from different interactive components [20].

Our study limitations are related to the small number of participants, the lack of involvement of the partners in the survey, and the limited time in which it was conducted. The type of recruitment can also be a limitation because the medical facility where the patients were recruited is a top one and there could be other aspects that are overlooked in different medical units. As we previously observed, the subjectivity of a mother’s answer may also be a determinant factor that has to be considered in the accuracy of results [21,22].

Another weak point of the study is represented by the heterogeneity of the study group regarding the level of education of the patients and the age of their children. It would be more relevant if mothers completed the questionnaire at the same period after birth.

We intend to expand the research in that area in order to reveal the most interesting topics for pregnant women and expecting couples in our cultural context (prenatal care, birth and labour, baby care and safety, mother wellbeing, mother mental health, and, last but not least, how to cope with infectious transmitted disease during pregnancy and after birth). The strength of the study is that it represents a mirror, regarding the perception of prenatal education in the Romanian population and aims to contribute to finding ways to optimize this important aspect of prenatal care.

## 5. Conclusions

Participants who attended the prenatal courses were aware of the importance of prenatal education. This is proven by the fact that even after the birth they almost unanimously appreciated the usefulness of these courses. The benefits of prenatal education were recognized by those women through their appreciation of the topics of the lecture. Women who did not participate underestimate the utility of the prenatal courses, although some topics were indicated as interesting by more than half of this category of respondents. Further actions are required to inform mothers about the necessity of antenatal education.

## Figures and Tables

**Table 1:** The utility of topics from the questionnaire.

	Topic from the Questionnaire	The Utility of Information (the Affirmative Answer)	
		Study Group (Percent, Number)	Control Group (Percent, Number)
1	Partner’s participation or intention to attend the course	90.3% (*n* = 56)	55.6% (*n* = 15)
2	Newborn care	93.5% (*n* = 58)	55.6% (*n* = 15)
3	Newborn feeding	90.3% (*n* = 56)	37% (*n* = 10)
4	Breastfeeding	90.3% (*n* = 56)	3.7% (*n* = 1)
5	Birth preparation	69.4% (*n* = 43)	14.8% (*n* = 4)
6	Mother alimentation and hygiene	51.6% (*n* = 32)	29.6% (*n* = 8)
7	Partner’s support	91.9% (*n* = 57)	88.9% (*n* = 24)
8	The mother’s help after 6 weeks from delivery	50.0% (*n* = 31)	48.1% (*n* = 13)
9	The mother’s help with breast feeding	83.9% (*n* = 52)	74.1% (*n* = 20)
10	The mother’s help with newborn care	95.2% (*n* = 59)	96.3% (*n* = 26)

## Data Availability

The data presented in this study are available on request from the corresponding author. The data are not publicly available due to patients’ privacy.
